# Supplementary material for: Effects of reflective warning markers on wildlife
Source: PeerJ. 2019 Sep 3;7:e7614. doi: 10.7717/peerj.7614 (PMC6730532; doi:10.7717/peerj.7614)
Supplement: Appendix S1 [file peerj-07-7614-s001.docx]

**Appendix**

Appendix 1

The list of species captured by camera traps

| Species | No. independent photography | CITES | No. captured camera | |
| --- | --- | --- | --- | --- |
|  |  |  | C | W |
| ⅠSCANDENTIA |  |  |  |  |
| 1 Tupaiidae |  |  |  |  |
| （1）*Tupaia belangeri* | 14 | Ⅱ | 2 | 1 |
| ⅡRODENTIA |  |  |  |  |
| 2 Sciuridae |  |  |  |  |
| （2）*Callosciurus erythraeus* | 168 |  | 10 | 7 |
| （3）*Dremomys pernyi* | 380 |  | 11 | 7 |
| （4）*Hystrix brachyura* | 2 |  |  | 1 |
| ⅢLAGOMORPHA |  |  |  |  |
| 3 Leporidae |  |  |  |  |
| （5）*Lepus comus* | 2 |  |  | 1 |
| ⅣARTIODACTYLA |  |  |  |  |
| 4 Suidae |  |  |  |  |
| （6）*Sus scrofa* | 70 |  | 9 | 5 |
| 5 Cervidae |  |  |  |  |
| （7）*Muntiacus muntjak* | 2 |  | 1 |  |
| （8）*Naemorhedus caudatus* | 30 |  | 3 | 1 |
| ⅤCARNIVORA |  |  |  |  |
| 6 Felidae |  |  |  |  |
| （9）*Prionailurus bengalensis* | 20 | Ⅱ | 4 | 2 |
| 7 Viverridae |  |  |  |  |
| （10*Paguma larvata* | 14 | Ⅲ | 3 |  |
| 8 Mustelidae |  |  |  |  |
| （11）*Martes flavigula* | 6 |  | 3 | 1 |
| Ⅶ GALLIFORMES |  |  |  |  |
| 9 Phasianidae |  |  |  |  |
| （12）*Chrysolophus amherstiae* | 178 |  | 13 | 9 |
| （13）*Tragopan temminckii* | 2 |  | 1 |  |
| Ⅷ STRIGIFORMES |  |  |  |  |
| 10 Strigidae |  |  |  |  |
| （14）*Strix aluco* | 1 |  |  | 1 |
| ⅨPASSERIFORMES |  |  |  |  |
| 11 Turdidae |  |  |  |  |
| （15）*Zoothera dixoni* | 2 |  |  | 1 |
| 12 Muscicapidae |  |  |  |  |
| （16）*Garrulax erythrocephalus* | 2 |  | 1 |  |
| （17）*Garrulax maximus* | 4 |  | 2 |  |
| （18）*Garrulax subunicolor* | 2 |  | 1 |  |
| （19）*Garrulax elliotii* | 2 |  | 1 |  |
| （20）*Garrulax cineraceus* | 2 |  |  | 1 |
| （21）*Pomatorhinus ruficollis* | 6 |  | 1 |  |
| （22）*Pomatorhinus erythrocnemis* | 5 |  | 1 |  |
| （23）*Niltava sundara* | 4 |  | 3 |  |
| （24）*Phoenicurus auroreus* | 1 |  | 1 |  |
| 13 Paridae |  |  |  |  |
| （25）*Aegithalos concinnus* | 2 |  | 1 |  |

Note: RWM is camera with RWM; C is control. CITES appendix I lists species that are the most endangered among CITES-listed animals; CITES appendix II lists species that are not necessarily now threatened with extinction but that may become so unless trade is closely controlled; CITES appendix III is a list of species included at the request of a Party that already regulates trade in the species and that needs the cooperation of other countries to prevent unsustainable or illegal exploitation. <https://www.cites.org/>
