# Supplementary material for: Effects of reflective warning markers on wildlife
Source: PeerJ. 2019 Sep 3;7:e7614. doi: 10.7717/peerj.7614 (PMC6730532; doi:10.7717/peerj.7614)
Supplement: Appendix S2 [file peerj-07-7614-s002.docx]

Appendix 2

Table. A2 The results of linear mixed model with treatments as fixed variates, sub-regions as the random variates, species number as response variates

| effects | | | | | | |
| --- | --- | --- | --- | --- | --- | --- |
| Random effects | Sub-regions | Name | Variance | Std.Dev |  |  |
|  | Date | (Intercept) | 0.1218 | 0.3489 |  |  |
|  | Residual |  | 5.4861 | 2.3422 |  |  |
| Fixed effects |  | Estimate | Std. Error | df | t value | Pr(>\|t\|) |
|  | (Intercept) | 2.6000 | 0.6246 | 12.5465 | 4.163 | 0.0012 ** |
|  | Treatment-Control | 2.3333 | 0.8553 | 23.9999 | 2.728 | 0.0117 * |
| Correlation of Fixed Effects | (Intr) |  |  |  |  |  |
|  | Treatment-Control 0.342 | |  |  |  |  |

* p < 0.05

** p < 0.01

Table. A3 The results of linear mixed model with treatments as fixed variates, sub-regions as the random variates, independent photographs number as response variates

|  | effects | | | | | |
| --- | --- | --- | --- | --- | --- | --- |
| Random effects | Sub-regions | Name | Variance | Std.Dev |  |  |
|  | Date | (Intercept) | 34.13 | 5.842 |  |  |
|  | Residual |  | 285.09 | 16.885 |  |  |
| Fixed effects |  | Estimate | Std. Error | df | t value | Pr(>\|t\|) |
|  | (Intercept) | 10.000 | 5.083 | 9.476 | 1.968 | 0.079 |
|  | Treatment-Control | 10.133 | 6.165 | 24.000 | 1.644 | 0.113 |
| Correlation of Fixed Effects | (Intr) |  |  |  |  |  |
|  | Treatment-Control -0.607 | |  |  |  |  |
